# Huge Cavernous Hemangiomas Enveloping the Optic Nerve Successfully Removed by a Vertical Lid Split Orbitotomy

**DOI:** 10.1155/2014/135252

**Published:** 2014-04-29

**Authors:** Jung-Hoon Yum, Yoon-Duck Kim, Jung Hye Lee, Kyung In Woo

**Affiliations:** ^1^Department of Ophthalmology, Inje University Ilsan Paik Hospital, 170 Juhwa-ro, Ilsanseo-gu, Goyang 411-706, Republic of Korea; ^2^Department of Ophthalmology, Samsung Medical Center, Sungkyunkwan University School of Medicine, 50 Irwon-dong, Gangnam-gu, Seoul 135-710, Republic of Korea; ^3^Department of Ophthalmology, Kim's Eye Hospital, Myung-Gok Eye Research Institute, Konyang University College of Medicine, 156 Yeongdeungpo-dong, Yeongdeungpo-gu, Seoul 150-034, Republic of Korea

## Abstract

A 63-year-old woman presented with a 15-year history of gradually increasing proptosis of right eye. Ocular examination revealed proptosis of 9 mm with decreased visual acuity in her right eye. CT scan showed a well-circumscribed and enhancing orbital mass filling almost the entire right orbit. The tumor occupied the superolateral, superomedial, and inferomedial intraconal space, enveloping the optic nerve. Complete excision of two large intraconal tumors was performed successfully via a vertical lid split orbitotomy. Histopathologic examination confirmed the diagnosis of cavernous hemangioma. There were no intraoperative or postoperative complications. The patient achieved a satisfactory cosmetic outcome 1 year after surgery.

## 1. Introduction

A lateral orbitotomy can be used as a primary approach for surgical removal of cavernous hemangioma located intraconally [1]. It is challenging to decide the appropriate surgical approach to remove large tumor when it is located in the medial intraconal space [2]. Approaches which have been described to excise large intraconal tumors include combined medial and lateral orbitotomy or circumferential pan-orbitotomy [3]. The current case illustrates successful excision of huge intraconal cavernous hemangiomas enveloping the optic nerve via a vertical lid split orbitotomy.

## 2. Case Report

**Figure 1:**
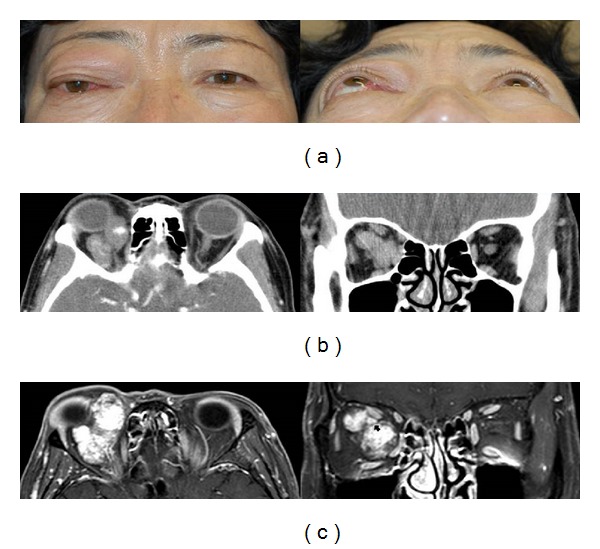
(a) Preoperative clinical photography shows the significant proptosis and inferolateral displacement of right eye. (b) CT scan of initial presentation shows well-demarcated enhancing intraconal vascular tumor in the right orbit. (c) 14 years later, MRI showing hypointense masses on T1-weighted sequence and hyperintense on T2-weighted sequence and demonstrating dense contrast enhancement. The optic nerve is surrounded and compressed by tumor (black arrow).

**Figure 2:**
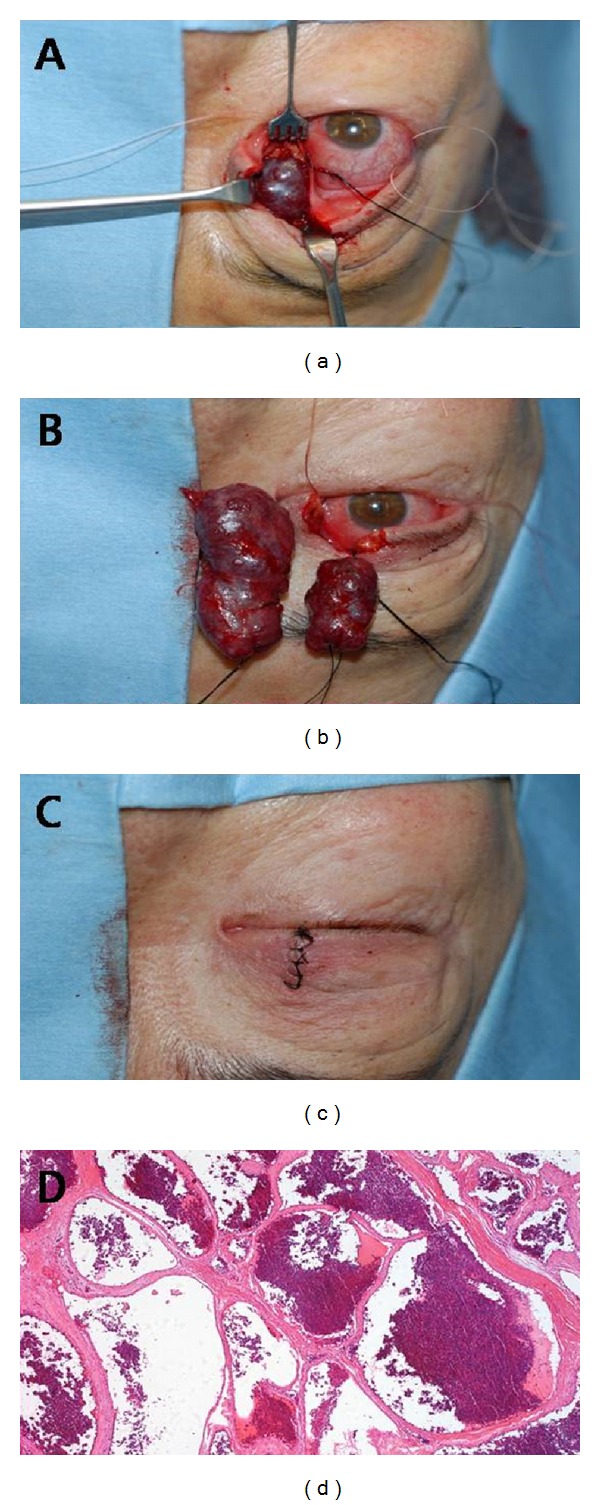
(a) Full thickness vertical lid marginal incision is made at the junction of the medial and central thirds of the upper lid. (b) After all adhesions were released carefully, the two tumors were removed successfully. (c) The lid incision was repaired by the same method used for repair of a full thickness lid margin laceration. (d) Histopathologic examination reveals cavernous hemangioma consisting of dilated spaces filled with blood and septated by fibrous tissues (hematoxylin-eosin, X50).

**Figure 3:**
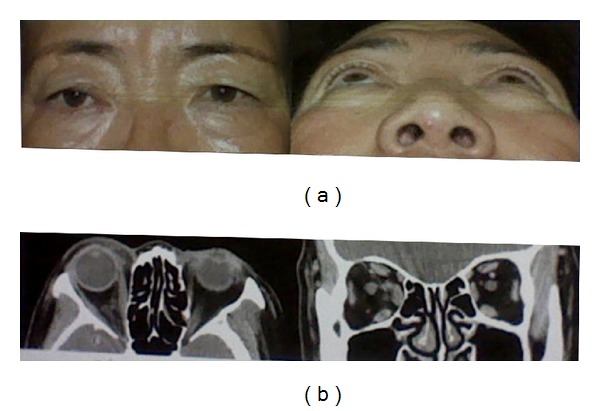
(a) Postoperatively, photographs showing that proptosis is improved with good cosmesis. (b) Postoperative CT scan demonstrating the complete removal of the tumors.

A 63-year-old female presented to our clinic with a painless and prominent right eye of 15 years duration (Figure 1(a)). When she first presented to us 14 years ago, she had no significant past medical history of note. Her visual acuity was 20/20 in both eyes. Hertel exophthalmometry measurement was 13.5 mm in the right eye and 10 mm in the left eye. Computed tomography (CT) scan showed well-demarcated, enhancing intraconal vascular tumor surrounding the optic nerve in the right orbit. After that time, she was lost to follow up. Two years ago, she revisited our clinic with progressive visual disturbance in her right eye. On ophthalmic examination, best corrected visual acuity was 20/200 OD and 20/20 OS. Hertel ophthalmometry revealed 9 mm proptosis of the right eye. There was a relative afferent pupillary defect and a color vision defect in the right eye. On fundus examination, she had mild elevation of the right optic disc. Goldmann visual field demonstrated a central and temporal scotoma of the right eye. Visual evoked potential (VEP) showed delay in P100 and decreased amplitude in the right eye. CT scan showed that the tumor occupied the superolateral, superomedial, and inferomedial intraconal spaces, enveloping the optic nerve and displaced the globe anteriorly and inferolaterally. The size of the tumor was larger as compared to 14 years ago (Figure 1(b)). MRI revealed the tumor was hypointense on T1-weighted sequence and hyperintense on T2-weighted sequence and demonstrated progressive filling on gadolinium enhanced sequences (Figure 1(c)). A vertical lid split orbitotomy was performed to remove the tumor. The larger tumor was firmly attached to the smaller tumor with fibrous band and the optic nerve was located between two tumors. Careful dissection was performed, so as not to damage the optic nerve. After all fibrous adhesions between two tumors were released, the larger tumor was removed successfully and then smaller tumor was removed safely. The lid incision was repaired with a similar method used for repair of a full thickness lid margin laceration. The masses measured 5 × 3 × 1 cm and 3 × 1 × 1 cm, respectively. Histopathologic examination confirmed the diagnosis of cavernous hemangiomas (Figure 2). There was no intraoperative complication, and the patient's recovery was uneventfully. At postoperative 6 months, best corrective visual acuity of the right eye was improved to 20/50. There was satisfactory cosmesis with no eyelid dysfunction. CT scan of the orbit showed no residual tumor in the orbit (Figure 3).

## 3. Discussion

Several surgical approaches for intraconal orbital tumor have been described in relation to the location and size of the lesion [1–3]. Lateral orbitotomy has been the preferred method to excise the cavernous hemangiomas as they are usually located intraconally and laterally. The removal of large cavernous hemangiomas in the medial intraconal space is challenging for complete excision without complications because visualization of tumor is limited and there are high density of critical normal structures often associated with substantial attachment to the tumor [2–4]. An anterior orbitotomy via an upper eyelid crease incision requires transection of the levator aponeurosis and Müller's muscle to reach to the intraconal space. In addition, disinsertion of medial rectus muscle is also needed in the medial approach. Transcranial approach or circumferential pan-orbitotomy is invasive procedure and spends much operative time [3].

Byron Smith reported the vertical lid split orbitotomy for removal of superonasal anterior orbital tumors [5]. Kersten and Kulwin described the detailed procedure of the vertical lid split approach [6]. This approach provides excellent exposure of the superomedial orbit and is a very useful approach to access lesions that lie in medial to the optic nerve [7].

In our case, the larger tumor was located at the superomedial part of the orbit and attached to another tumor laterally, enveloping the optic nerve. With its close proximity to the optic nerve, dissection of the tumors was challenging. We excised the tumors completely via vertical lid split orbitotomy and saved operation time. The patient recovered fast and had an uneventful postoperative period.

In conclusion, the huge intraconal cavernous hemangiomas could be safely removed via vertical lid split orbitotomy. Postoperatively, it maintains the normal eyelid function and yields good cosmetic outcome. It is an excellent alternative approach to extract large intraconal, well encapsulated tumor which is superomedially located in the orbit.
